# Substitutional semi-rigid osteosynthesis technique for treatment of unstable pubic symphysis injuries: a biomechanical study

**DOI:** 10.1007/s00068-023-02333-6

**Published:** 2023-08-09

**Authors:** Till Berk, Ivan Zderic, Peter Varga, Peter Schwarzenberg, Karlyn Berk, Niklas Grüneweller, Tatjana Pastor, Sascha Halvachizadeh, Geoff Richards, Boyko Gueorguiev, Hans-Christoph Pape

**Affiliations:** 1 AO Research Institute Davos, Clavadelerstrasse 8, 7270 Davos, Switzerland; 2 Department of Trauma, University Hospital Zurich, Raemistrasse 100, 8091 Zurich, Switzerland; 3 Harald-Tscherne Laboratory for Orthopedic and Trauma Research, University of Zurich, Sternwartstrasse 14, 8091 Zurich, Switzerland; 4 Department of Plastic and Hand Surgery, Inselspital University Hospital Bern, University of Bern, Freiburgstrasse 15, 3010 Bern, Switzerland; 5 Department of Trauma Surgery and Orthopedics, Protestant Hospital of Bethel Foundation, University Hospital OWL of Bielefeld University, Campus Bielefeld‑Bethel, Burgsteig 13, 33617 Bielefeld, Germany

**Keywords:** Symphysis injuries, Alternative fixation technique, Semi-rigid fixation, Biomechanics, Motion tracking, Karlyn method

## Abstract

**Background/Purpose:**

The surgical fixation of a symphyseal diastasis in partially or fully unstable pelvic ring injuries is an important element when stabilizing the anterior pelvic ring. Currently, open reduction and internal fixation (ORIF) by means of plating represents the gold standard treatment. Advances in percutaneous fixation techniques have shown improvements in blood loss, surgery time, and scar length. Therefore, this approach should also be adopted for treatment of symphyseal injuries. The technique could be important since failure rates, following ORIF at the symphysis, remain unacceptably high. The aim of this biomechanical study was to assess a semi-rigid fixation technique for treatment of such anterior pelvic ring injuries versus current gold standards of plate osteosynthesis.

**Methods:**

An anterior pelvic ring injury type III APC according to the Young and Burgess classification was simulated in eighteen composite pelvises, assigned to three groups (*n* = 6) for fixation with either a single plate, two orthogonally positioned plates, or the semi-rigid technique using an endobutton suture implant. Biomechanical testing was performed in a simulated upright standing position under progressively increasing cyclic loading at 2 Hz until failure or over 150,000 cycles. Relative movements between the bone segments were captured by motion tracking.

**Results:**

Initial quasi-static and dynamic stiffness, as well as dynamic stiffness after 100,000 cycles, was not significantly different among the fixation techniques (*p* ≥ 0.054).). The outcome measures for total displacement after 20,000, 40,000, 60,000, 80,000, and 100,000 cycles were associated with significantly higher values for the suture technique versus double plating (*p* = 0.025), without further significant differences among the techniques (*p* ≥ 0.349). Number of cycles to failure and load at failure were highest for double plating (150,000 ± 0/100.0 ± 0.0 N), followed by single plating (132,282 ± 20,465/91.1 ± 10.2 N), and the suture technique (116,088 ± 12,169/83.0 ± 6.1 N), with significantly lower values in the latter compared to the former (*p* = 0.002) and no further significant differences among the techniques (*p* ≥ 0.329).

**Conclusion:**

From a biomechanical perspective, the semi-rigid technique for fixation of unstable pubic symphysis injuries demonstrated promising results with moderate to inferior behaviour compared to standard plating techniques regarding stiffness, cycles to failure and load at failure. This knowledge could lay the foundation for realization of further studies with larger sample sizes, focusing on the stabilization of the anterior pelvic ring.

## Introduction

Pelvic ring injuries require a considerable spectrum of treatment options ranging from nonoperative therapies to necessary surgical stabilization in life-threatening situations. The surgical fixation of a symphyseal diastasis in partially or fully unstable pelvic ring injuries is an important element when stabilizing the anterior pelvic ring [1]. A vast selection of surgical stabilization techniques exists and ranges from fixations spanning the pubic symphysis and/or sacroiliac (SI) joints to open and minimally invasive percutaneous techniques [2, 3]. Open reduction and internal fixation (ORIF) of the pubic symphysis is a well-established procedure that has been performed for several decades, initially starting via cerclage wiring, then developing to external fixation, and eventually progressing to current techniques of plate and screw fixation [4–6]. Furthermore, the advancement toward percutaneous fixation of the symphysis has revealed significant improvements regarding intraoperative blood loss, surgery time, and scar length when compared to ORIF via plating [7–9]. However, even with these significant improvements in minimally invasive techniques, percutaneous closed reduction and internal fixation (CRIF) of anterior pelvic ring injuries has resulted in failure rates of up to 15% [8–10]. These techniques have further been associated with higher rates of failure when compared to percutaneous fixations of posterior pelvic ring injuries [8–10]. Regarding ORIF of the anterior pelvic ring, there is a high prevalence of anterior plate failures in up to 21% of the cases [1, 11–13]. These unacceptably high numbers of implant failure suggest that plate osteosynthesis could result in a too rigid construct for this mobile anatomical region.

### Purpose

The aim of this biomechanical study was to evaluate an alternative semi-rigid fixation technique for treatment of anterior pelvic ring injuries versus the current gold standards of plate osteosynthesis [1, 14]. This technique was designed to provide a decrease in the rates of failure and complications, and to avoid or simplify secondary operations for implant removal (IR) within the spectrum of minimally invasive pelvic surgery. It was hypothesized that the alternative fixation technique using an endobutton suture implant [15] could achieve comparable stability to the standards of plate osteosynthesis.

## Materials and methods

### Specimens and preparation

Eighteen composite pelvises (Model LSS4060/Hard®, Synbone, Zizers, Switzerland) were used. An anterior pelvic ring injury type III APC according to the Young and Burgess classification [16] was simulated by cutting the stabilization material of the symphysis prior to instrumentation of the SI joint, resulting in a pubic diastasis of more than 5 cm and leading to a global instability.

The eighteen specimens were assigned to three groups (*n* = 6) for instrumentation as follows:

- Group Symphysis Single Plate (SSP)—fixation of the pelvic symphysis with a Curved Pelvic Plate, Radius 88, 4 holes (Stryker, Selzach, Switzerland).
- Group Symphysis Double Plate (SDP)—fixation of the pelvic symphysis with a Curved Pelvic Plate, Radius 88, 4 holes (Stryker, Selzach, Switzerland) and an additional anteriorly placed 5-hole 3.5 mm Locking Compression Plate (LCP; DePuy Synthes, Zuchwil, Switzerland).
- Group Symphysis TightRope® (STR)—fixation of the pelvic symphysis with an anteriorly placed AC TightRope® Twin Tail (Arthrex, Munich, Germany) applied through four drill holes.

A priori power analysis resulted in a minimum of six specimens per group for statistical power of 0.8 at a level of significance 0.05 under the assumption that the standard deviation in each group is not bigger than 60% of the minimum difference in mean values between the groups.

The plates and screws in groups SSP and SDP were made of stainless steel 316LVM. Prior to instrumentation, each pelvis was discontinued by cutting the pubic ramus on the right side at mid-shaft and removing the sacrum, whereas the left hemi-pelvis was left unaffected.

The symphysis of each specimen was anatomically reduced prior to instrumentation.

In group SSP, the plates and screws, specifically designed for applications in the pubic symphysis, were placed according to the AO Surgery Reference [17] and the manufacturer's recommendations. Four bicortical screws of appropriate lengths were placed under direct visualization to achieve satisfactory fixation. The screws were inserted exclusively inside the bone, avoiding ventral and dorsal cortical perforations as well as symphysis joint penetrations.

The additional plate in group SDP was placed ventrally directly below the cranial edge of the symphysis. Two 3.5 mm fully threaded cortical screws were inserted in the left and right segments of the symphysis, avoiding conflicts with the screws already inserted in cranial–caudal direction.

**Fig. 1:**
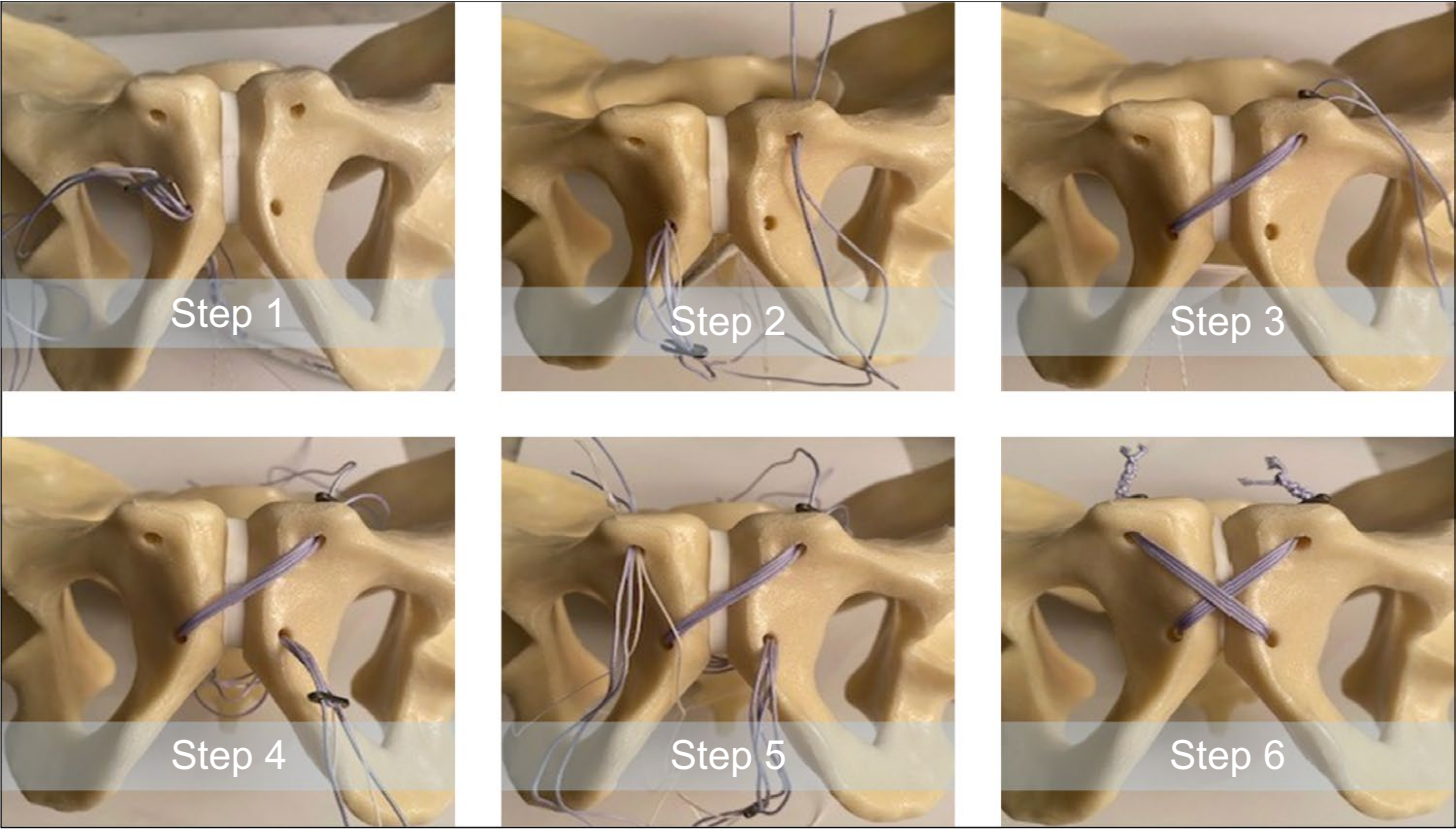
Surgical procedure for TightRope^®^ fixation applying the Karlyn method

Prior to the implant fixation in group STR, two 4.5 mm bicortical holes were drilled in the superior surface of each superior pubic ramus at a distance of 2 cm lateral from the pubic symphysis and directed from superior to anterior in a 15° angle. Then, two 4.5 mm bicortical holes were drilled in the cranial portion of each inferior pubic ramus, placed 1 cm lateral to the pubic symphysis and 4 cm below its cranial edge and directed from anterior to posterior. Next, the accompanying nitinol suture was used to pass the suture/endobutton through the inferior right hole from anterior to posterior and then cranially to the superior left hole. Further, the nitinol suture was used again to pass the suture/endobutton from the inferior right hole over to the inferior left hole and then cranially to the superior right hole from anterior to posterior. Two endobuttons came to rest on the cranial surface of the two superior pubic rami, while the third endobutton came to rest dorsally on the inner side of the right hole of the inferior pubic ramus. The sutures were then finally tightened in place by constant traction and surgical knotting at the two superior endobuttons. This technique, named Karlyn method, is illustrated in Fig. 1.

**Fig. 2:**
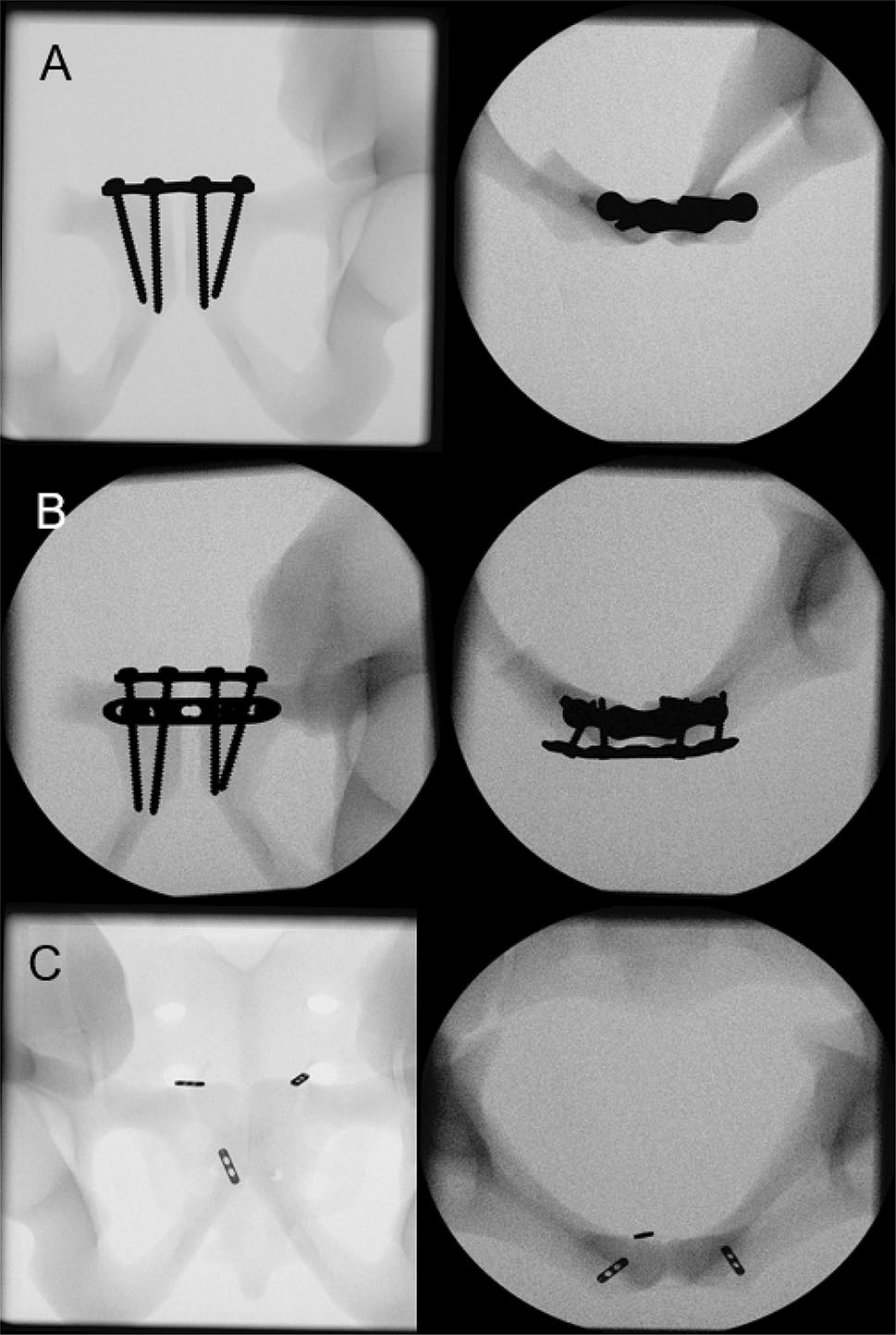
Anteroposterior (left) and axial (right) X-rays after instrumentation visualizing exemplified specimens from group SSP (**A**), SDP (**B**), and STR (**C**)

An experienced surgeon with senior consultant status performed all procedures. After instrumentation, anteroposterior and axial X-rays were performed for documentation and verification of the positioning of the plates, screws and endobuttons (Fig. 2).

## Biomechanical testing

**Fig. 3:**
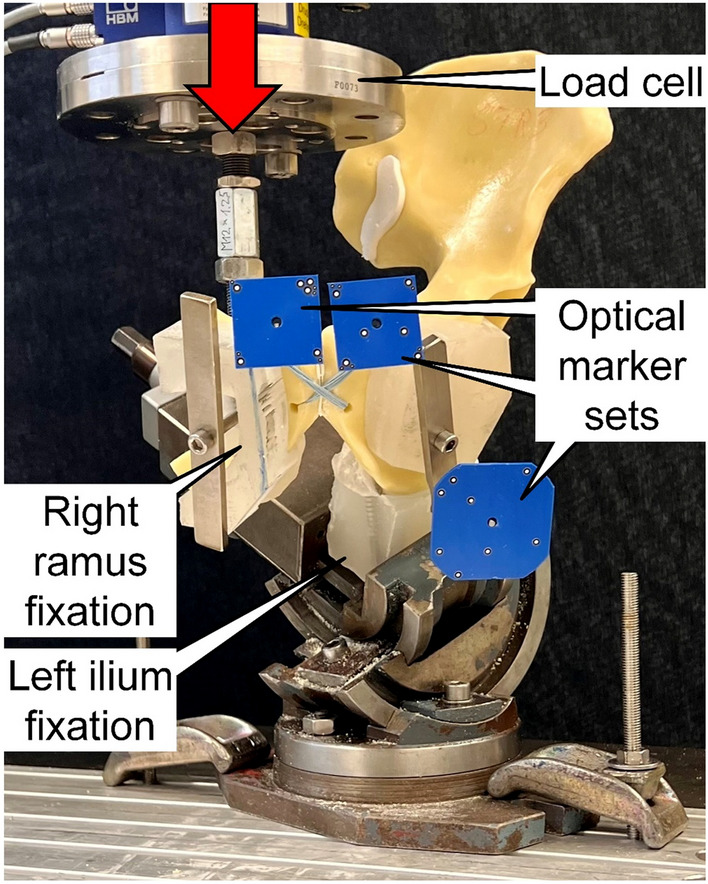
Test setup with a specimen mounted for biomechanical testing. Vertical arrow denotes loading direction

Biomechanical testing was performed on a servohydraulic material testing system (Bionix 858.20; MTS Systems, Eden Prairie, MN, USA) equipped with a 5 kN/50 Nm load cell. The setup with a specimen mounted for testing is shown in Fig. 3. Each specimen was aligned and tested in a simulated standing upright position. The ilium of the left anatomical side was constrained to the machine base via a vice, fixing the portion of the ilium between the ramus and the proximal border of the acetabulum. Two custom polymethylmethacrylate (PMMA) blocks were molded for repeated use and application of a homogeneous clamping force exerted by the vice. The holding strength of the PMMA blocks was enhanced via a steel bar connection via a transfixing screw. Loading along the machine axis was applied via the machine transducer to the right superior ramus. To achieve this, a custom PMMA block was connected to the machine transducer via an embedded threaded steel rod and attached to the right ramus via a steel bar. Three retro-reflective marker sets were attached to the cranial aspect of each pubic symphysis side and to the ilium PMMA block for optical motion tracking. The plane of the ilium marker set was aligned with the coronal plane of the specimens.

The loading protocol commenced with an initial nondestructive compressive quasi-static ramp from 0 to a 25 N preload at a rate of 2.5 N/s, followed by progressively increasing cyclic loading in both axial tension and compression with a physiological profile of each cycle at a rate of 2 Hz [19]. The peak load of both tension and compression monotonically increased cycle by cycle at a rate of 0.0005 N/cycle until catastrophic failure of the specimen [20, 21] or over 150,000 test cycles. The loading protocol was tuned in a priori pilot study so that a targeted number of minimum 55,500 cycles would be reached for each specimen, reflecting the reported number of cycles absolved by patients who received hip endoprosthesis within a post-operative rehabilitation period of 6 weeks [22].

## Data acquisition and analysis

Machine data in terms of axial displacement and axial load were continuously acquired from the machine transducer and load cell throughout the tests at 200 Hz. Based on these data, construct stiffness was calculated from the ascending load–displacement curve of the initial quasi-static ramp—defined as initial quasi-static  stiffness—and then from the 3rd and the 100,000th test cycles—the latter two defined as initial dynamic stiffness and dynamic stiffness after 100,000 cycles, respectively. Furthermore, the dynamic stiffness after 100,000 cycles was normalized to the initial dynamic stiffness to evaluate the relative change over time.

The positions of the optical markers were continuously recorded at 20 Hz during testing from a stereographic camera system (Aramis SRX, Carl Zeiss GOM Metrology GmbH, Braunschweig, Germany). The motion tracking data were used to evaluate the magnitude of the intersegmental movements at the most inferior aspect of the pubic symphysis—defined as total displacement. The outcome measures of this parameter of interest were analyzed after 20,000, 40,000, 60,000, 80,000, and 100,000 cycles under peak compressive loading with respect to the beginning of the cyclic test. In addition, a clinically relevant criterion for specimen's failure was arbitrarily set at 2 mm total displacement according to previous findings by Walheim et al. [23] who reported that symphyseal movements in vertical direction amounted up to 2 mm. Correspondingly, the number of cycles until fulfillment of this criterion—defined as cycles to failure—was calculated together with the respective peak compressive load—defined as load at failure.

Statistical analysis was performed with SPSS software (v.27, IBM SPSS, Armonk, NY, USA). Normality of data distribution was screened and proved with Shapiro–Wilk test. Mean value and standard deviation (SD) were calculated for each parameter of interest and group separately. One-Way Analysis of Variance (ANOVA) and General Linear Model Repeated Measures tests with Bonferroni post-hoc test for multiple comparisons were conducted to detect significant differences among the groups. Level of significance was set at 0.05 for all statistical tests.

## Results

**Fig. 4:**
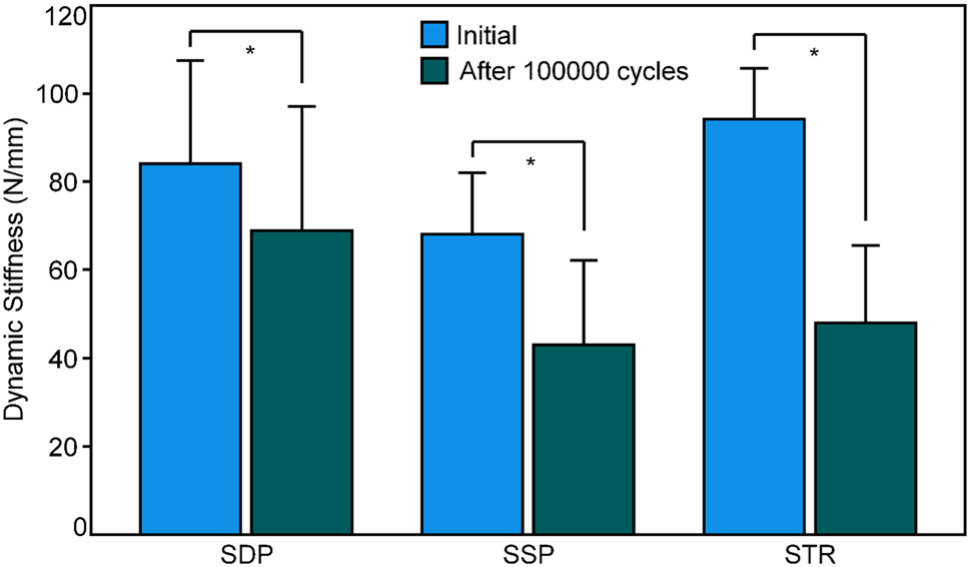
Initial dynamic stiffness and dynamic stiffness after 100,000 cycles, shown in terms of mean value and SD for each group separately. Stars indicate significant differences

Outcome measures for initial quasi-static and dynamic stiffness, as well as for dynamic stiffness after 100,000 cycles, are presented in Table 1. No significant differences were detected among the groups for any of these investigated parameters (*p* ≥ 0.054). However, the drop in dynamic stiffness after 100,000 cycles relative to the initial dynamic stiffness was significant in each group (*p* ≤ 0.003; Fig. 4). Furthermore, this drop was biggest in group STR (49.7 ± 12.1% (mean ± SD)), followed by group SSP (38.9 ± 15.9%) and group SDP (20.9 ± 13.2%), and in addition it was significantly bigger in group SSP versus group SDP (*p* = 0.009), with no further significant differences between the pairs of groups (*p* ≥ 0.127).

**Table 1:** Outcome measures for initial quasi-static, initial dynamic, and dynamic stiffness after 100,000 cycles, presented for each group separately in terms of mean value and SD, together with *p*-values from the statistical evaluation among the groups

Parameter of interest		Group	Mean (SD)	*p*-value
Stiffness (N/mm)	Initial quasi-static	SDP	82.9 (20.2)	0.061
		SSP	73.6 (17.2)	
		STR	98.4 (11.4)	
	Initial dynamic	SDP	84.0 (23.4)	0.054
		SSP	68.1 (13.9)	
		STR	94.2 (11.6)	
	Dynamic after 100,000 cycles	SDP	68.9 (28.2)	0.133
		SSP	43.0 (19.1)	
		STR	48.1 (17.4)

**Fig. 5:**
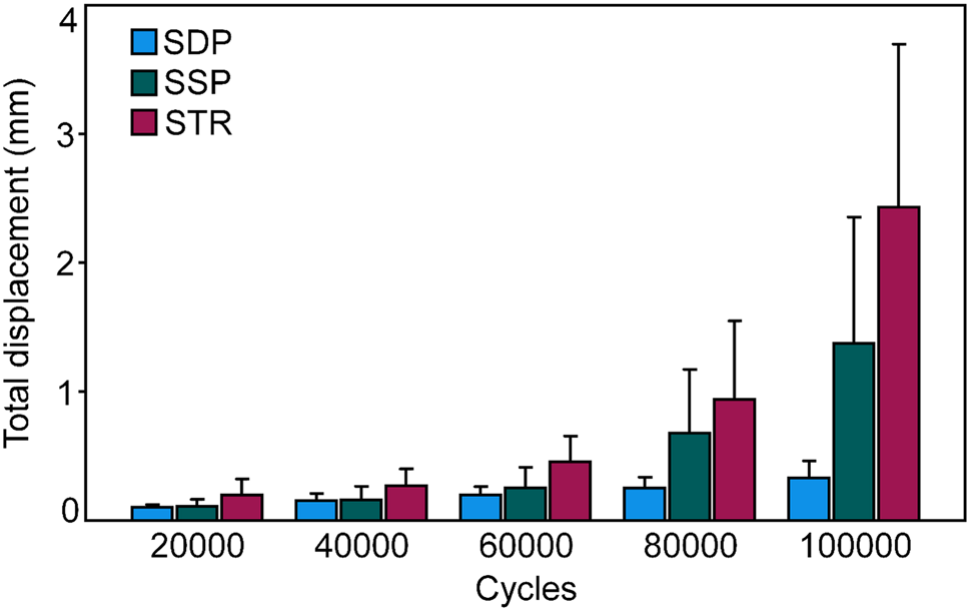
Total displacement after 20,000, 40,000, 60,000, 80,000, and 100,000 test cycles, shown for each group separately in terms of mean value and SD

The outcome measures for total displacement, analyzed over the five time points after 20,000, 40,000, 60,000, 80,000, and 100,000 cycles are shown in Fig. 5. They were associated with significantly higher values in group STR versus group SDP (*p* = 0.025), without further significant differences detected between the pairs of groups (*p* ≥ 0.349).

**Fig. 6:**
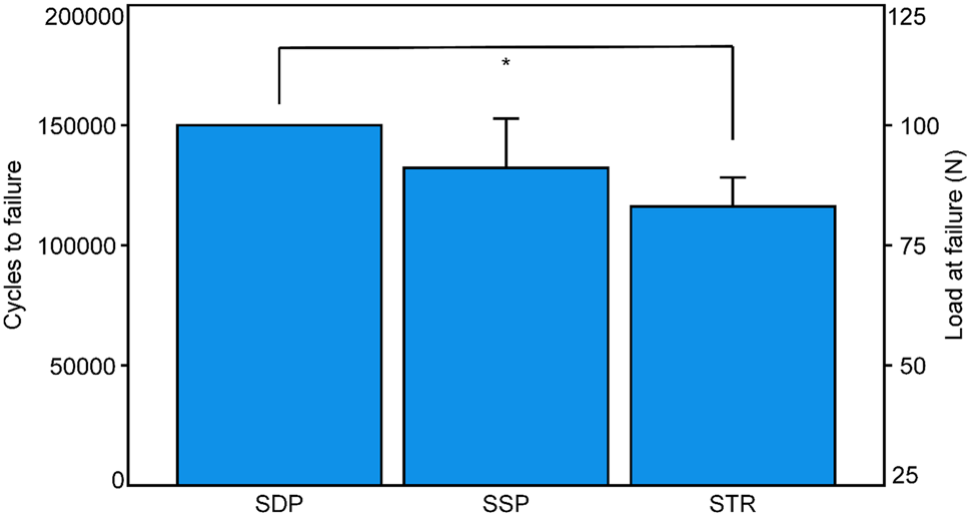
Cycles to failure and load at failure shown for each group separately in terms of mean value and SD. Star indicates significant difference

Cycles to failure and load at failure were highest in group SDP (150,000 ± 0/100.0 ± 0.0 N), followed by group SSP (132,282 ± 20,465/91.1 ± 10.2 N), and group STR (116,088 ± 12,169/83.0 ± 6.1 N), with significantly lower values in group STR compared to group SDP (*p* = 0.002) and without further significant differences between the pairs of groups (*p* ≥ 0.329; Fig. 6).

All except one specimen from group SDP and two specimens from group SSP survived 150,000 test cycles without catastrophic failure. The catastrophic failure modes were predominantly expressed by fracturing of the superior and inferior pubic ramus, equally on both sides, and located between the fixation to the machine base and the implants.

## Discussion

The aim of this biomechanical study was to assess the stability of a novel semi-rigid technique for stabilization of pubic symphysis injuries using an endobutton suture implant.

With regard to the biomechanical testing results, the following three main important points were identified:

1. The initial quasi-static and dynamic stiffness was comparable between all groups.
2. Regarding the number of cycles to 2 mm total displacement, group STR demonstrated significantly lower values when compared to group SDP.
3. All specimens in group STR exclusively reached the maximum number of test cycles without catastrophic failure.

Open reduction and plate fixation via the Pfannenstiel approach is currently the leading therapy of symphyseal injuries [24]. However, this surgical approach poses disadvantages, such as dissections through a dense anatomic region, high blood loss, long surgery time, and potential injury of surrounding structures. Iatrogenic injuries to the inguinal canal and its structures can result in remaining pain symptoms, and injuries to the corona mortis are known for devastating hemorrhages [24]. Since the symphysis is considered a synarthrosis with a physiological movement of up to 2 mm, a rigid plate fixation, considered as current gold standard treatment, can potentially result in implant failure and revision surgeries [14, 23, 25–27]. It was already proven in the literature that osteosyntheses of the anterior pelvic ring are known for a high prevalence of anterior plate failures [1, 11–13, 28]. Although multi-hole plates have proven to be a better option than two-hole plates, the failure rate remains high [11]. Alternative implants, such as external fixator or Infix used for anatomic reduction and fixation of the pubic symphysis, are known to be difficult to achieve reasonable stability due to the large distance from the fixation points to the symphysis [24].

The literature is controversial regarding the stability of different fixation methods. A previous study revealed that plate fixation is not necessarily superior versus cannulated screw fixation of the symphysis [29]. Another work comparing percutaneous screw fixation versus open reduction and plate fixation of the symphysis reported no significant differences in implant failure, wound infection, or revision surgery [7]. Yao et al. conducted a biomechanical finite element analysis comparing different methods of symphyseal fixations [29]. One of their findings was that dual screw fixation was stiffer than both plating constructs. The combined anterior and posterior fixation has been shown to achieve a decrease in the number of plate failures from 40 to 5% in type APC-2 pelvic injuries [1]. These findings are comparable to the results from the current study. While the initial quasi-static and dynamic stiffness was highest for STR, no significant differences between the groups could be detected. The results of both these studies may indicate that, in addition to standard plate osteosynthesis, very high initial stability can be achieved using minimally invasive methods. Since the analyzed injury was ligamentous, when the enclosed structure of the pelvic ring is restored by surgical repair, walking distance limitation could be sufficient to serve as a criterion for success of a treatment option in the future.

Semi-rigid implants, similar to the one used in this study, have been proven to avoid an unphysiological arthrodesis of the symphysis joint without influencing the ligamental healing [30].

The initial stiffness in the present work was statistically comparable among the groups but revealed higher mean values for STR versus SDP, which can be perceived as counterintuitive. This could be interpreted by the narrow load margin of 10–20 N within which the evaluation was performed, and within which the constructs may not have had the chance to settle. However, our results showed that the dynamic stiffness dropped most after 100,000 cycles in group STR, significantly more when compared to group SDP, thus allowing a physiological movement of the symphysis during the simulated walking. A previous study has reported that under physiologic loading conditions the forces and moments acting in the symphysis during the mobilization phase amount to 169 N in vertical direction, 68 N along the sagittal axis, and up to 2.5 Nm in the sagittal plane [31]. These values are somewhat higher than the failure loads obtained in the present study. Hence, combined with the achieved physiologically relevant numbers of cycles to failure, the applied loading protocol was deemed appropriate.

While the results for group STR are largely comparable to the standard of care in many aspects, three main advantages could come into effect applying the surgical treatment using the Karlyn method. The first advantage could be the option for a minimally invasive approach, even possibly endoscopically. The reduction of the symphysis can hereby be achieved with manual pressure on both ileums, following final reduction with a percutaneously inserted reduction clamp anteriorly, through the pre-existing inferior incisions as well as through the tightening of the endobuttons implant itself. The second advantage could be a possible omission of secondary implant removal. The semi-rigid fixation in group STR interferes much less with the bones and contains significantly less material, which potentially provides a target for irritation. The last potential advantage is in regards to the possibility of removal of the implant itself. If indicated at all, this could be much simpler compared to plate osteosynthesis with no risk of broken screws.

Only one clinical study and two biomechanical studies have, to our knowledge, been published, analyzing suture fixations of the pubic symphysis. A biomedical cadaveric study compared a suture fixation to plating and concluded that the suture button fixation was biomechanically similar to plate fixation [32].

A clinical study compared a TightRope® augmentation of an external fixator treatment versus percutaneous cannulated screw fixation of the pubic symphysis in unstable pelvic ring injury. This study also reported no significant differences between the treatment options [33].

The most recent study from 2021 compared plating of the pubic symphysis versus modified SpeedBridge™ in criss-cross/triangle technique with no significant difference between all of the compared osteosynthesis methods [34].

Numerous patients require implant removal due to discomfort, psychological or obstetric reasons, or in prevention of possible plate-associated complications [35]. Our personal experience with the AC TightRope® Twin Tail considering treatment of ligamentous injuries surrounding the clavicle has shown that the implant components grow into the surrounding tissue. After healing, they usually remain in place, even if the suture is torn and could be removed from its initial position.

To our knowledge, the presented Karlyn method has not been described before and since this study was performed on artificial bones, it is difficult to make statements for potential surgical problems in situ. Conceivable complications of the method could be fractures in the area of the drill holes due to cutting of the suture into the bone. In addition, the drill holes that are not plugged with a screw could lead to postoperative bleeding and thus to a hematoma. Furthermore, for a possible complete implant removal, as it may be necessary in the event of an infection, it could be difficult to recover the material completely via the minimally invasive approaches. The injuries studied are typically a result of a high-velocity trauma, which is often associated with a severe soft-tissue trauma. Therefore, it remains unclear whether a minimally invasive surgical technique in the impact zone can be applied at all. Finally, it remains unclear whether the symphysis can heal at all with the Karlyn-method, as it is the case with a plate.

A potential clinical concern regarding the use of suture button fixation was previously raised claiming that implant-related irritation of the bladder could occur [32]. The double-plate approach of the symphysis, as biomechanically tested in this study, has already been clinically established [36–38]. The authors are not aware of any indication in the literature that the bicortical screws which are in contact inside of the pelvis in the same way as the endobuttons, have caused irritation. In addition, in the technique described in this study, only a single endobutton was placed inside of the pelvis, compared to at least 4 bicortical screws used for the double-plate approach.

The results of this study demonstrated from a biomechanical perspective that the technique with TightRope® suturing is comparable to the standard single plating and inferior to double-plate osteosynthesis. As a clinically translated consequence of this, the patients may be forced to undergo a less aggressive rehabilitation protocol. Hence, stronger and less compliant semi-rigid constructs would be preferred, but have yet to be designed. Possible advantages of the TightRope® suturing in the context of symphyseal rupture fixation include the following aspect. The trans-symphyseal screw fixation can be considered as problematic, since it results in an iatrogenic damage to the symphysis itself. The consequences of this damage have not yet been adequately clarified according to current literature. Due to the minimally invasive lacing, the symphyseal structures are not affected. The same also applies to plate osteosynthesis, despite the known limitations.

### Strength and limitations

The main limitation of this study is the use of synthetic specimens where, due to the lack of ligaments, a less physiological condition is reproduced. This circumstance makes the simulation of a ligamentous injury to the pelvis difficult. However, as this is an experimental study with a novel approach, with little data available, the authors have chosen this study design as a first step. Only after a successful first step investigation, it is, to our opinion, ethically justifiable, to proceed with a cadaveric study in the second step. Further, to accommodate this limitation, a type III APC injury according to the Young and Burgess classification was deliberately chosen. According to its definition, the ligaments are ruptured, and an absolutely unstable situation prevails. Therefore, the ligaments could be largely neglected in the selected test series.

Additionally, it is known, that artificial pelvises allow a standardized study group, which overpowers the incalculable variations in bone quality that is possible when using human cadaveric specimens [39–41]. Furthermore, synthetic bone specimens are frequently and effectively used in various biomechanical studies, explicitly with focus on the pelvis [39, 42–45]. Additionally, the increasingly poor availability of cadavers can limit the sample size for biomechanical investigations and it is known that the sample sizes used in previous publications are generally small [46]. Also, the use of artificial bone models minimizes the variability of test results between test samples [44, 47]. In this regard, consistency of instrumentation was ensured to the extent that one experienced surgeon performed all procedures according to his discretion. Although screw and suture tightening were not quantitatively measured, the relatively small data scattering justifies this hands-on approach. In addition, this study cannot answer the question of whether closed reduction by manual compression of the pelvis is sufficient to provide the necessary reduction of injury for the minimally invasive treatment. Finally, the chosen sample size was relatively small, yet retrospectively sufficient considering the ability to detect significant differences between the groups and was in addition comparable to similar biomechanical studies investigating pelvic fixation techniques [42–45, 48].

## Conclusion

From a biomechanical perspective, the semi-rigid technique for fixation of unstable pubic symphysis injuries, using the Karlyn method with endobutton suture implants, demonsrated promising results with moderate to inferior behaviour compared to standard plating techniques regarding stiffness, cycles to failure, and load at failure. This knowledge could lay the foundation for realization of  further studies with larger sample sizes, focusing on the stabilization of the anterior pelvic ring, as well as for development of specifically designed stronger and more robust implants which could provide evidence to establish minimally invasive and endoscopic surgical therapies for stabilization of symphysis ruptures in future.

## Data Availability

The collected data will be stored securely for 10 years. During this period, they are still available upon request. After 10 years, the data will be deleted, however, all the datasets analyzed or generated during this study will be available from the corresponding author upon reasonable request.
